# Supplementary material for: Safety, efficacy and health impact of electronic nicotine delivery systems (ENDS): an umbrella review protocol
Source: BMJ Open. 2024 Jan 29;14(1):e080274. doi: 10.1136/bmjopen-2023-080274 (PMC10826537; doi:10.1136/bmjopen-2023-080274)
Supplement: Supplementary data [file bmjopen-2023-080274supp001.pdf]

Supplementary materials

Supplementary table 1: PRISMA-P

Section and topic	Item No	Checklist item	Location
**ADMINISTRATIVE INFORMATION**			
Title:			
Identification	1a	Identify the report as a protocol of a systematic review	Page 1 (Umbrella review)
Update	1b	If the protocol is for an update of a previous systematic review, identify as such	N/A
Registration	2	If registered, provide the name of the registry (such as PROSPERO) and registration number	Page 4*
Authors:			
Contact	3a	Provide name, institutional affiliation, e-mail address of all protocol authors; provide physical mailing address of corresponding author	Page 1
Contributions	3b	Describe contributions of protocol authors and identify the guarantor of the review	Page 7
Amendments	4	If the protocol represents an amendment of a previously completed or published protocol, identify as such and list changes; otherwise, state plan for documenting important protocol amendments	Page 4
Support:			
Sources	5a	Indicate sources of financial or other support for the review	Page 7
Sponsor	5b	Provide name for the review funder and/or sponsor	Page 7
Role of sponsor or funder	5c	Describe roles of funder(s), sponsor(s), and/or institution(s), if any, in developing the protocol	Page 7
**INTRODUCTION**			
Rationale	6	Describe the rationale for the review in the context of what is already known	Page 3
Objectives	7	Provide an explicit statement of the question(s) the review will address with reference to participants, interventions, comparators, and outcomes (PICO)	Page 3,4
**METHODS**			
Eligibility criteria	8	Specify the study characteristics (such as PICO, study design, setting, time frame) and report characteristics (such as years considered, language, publication status) to be used as criteria for eligibility for the review	Page 4,5, table 1
Information sources	9	Describe all intended information sources (such as electronic databases, contact with study authors, trial registers or other grey literature sources) with planned dates of coverage	Page 5
Search strategy	10	Present draft of search strategy to be used for at least one electronic database, including planned limits, such that it could be repeated	Table 2
Study records:			
Data management	11a	Describe the mechanism(s) that will be used to manage records and data throughout the review	Page 7

Goel S, *et al. BMJ Open* 2024; 14:e080274. doi: 10.1136/bmjopen-2023-080274

Selection process	11b	State the process that will be used for selecting studies (such as two independent reviewers) through each phase of the review (that is, screening, eligibility and inclusion in meta-analysis)	Page 7
Data collection process	11c	Describe planned method of extracting data from reports (such as piloting forms, done independently, in duplicate), any processes for obtaining and confirming data from investigators	Page 7 Supplementary table 2
Data items	12	List and define all variables for which data will be sought (such as PICO items, funding sources), any pre-planned data assumptions and simplifications	Page 7 Supplementary table 2
Outcomes and prioritization	13	List and define all outcomes for which data will be sought, including prioritization of main and additional outcomes, with rationale	Page 7
Risk of bias in individual studies	14	Describe anticipated methods for assessing risk of bias of individual studies, including whether this will be done at the outcome or study level, or both; state how this information will be used in data synthesis	Page 8
Data synthesis	15a	Describe criteria under which study data will be quantitatively synthesized	Page 8
	15b	If data are appropriate for quantitative synthesis, describe planned summary measures, methods of handling data and methods of combining data from studies, including any planned exploration of consistency (such as I^2, Kendall's τ)	Page 78
	15c	Describe any proposed additional analyses (such as sensitivity or subgroup analyses, meta-regression)	Page 78
	15d	If quantitative synthesis is not appropriate, describe the type of summary planned	Page 8
Meta-bias(es)	16	Specify any planned assessment of meta-bias(es) (such as publication bias across studies, selective reporting within studies)	Page 8
Confidence in cumulative evidence	17	Describe how the strength of the body of evidence will be assessed (such as GRADE)	Page 9

*Pending

Supplementary table 2: pre-piloted data extraction form

Doi	
Author and Year	
Objectives	
Details of sources searched	
Type of included studies	

Goel S, *et al. BMJ Open* 2024; 14:e080274. doi: 10.1136/bmjopen-2023-080274

No of included studies	
Country of included studies	
Participants (characteristics/total number)	
Description of Intervention	
Range (years) of included studies	
Quality assessment tool used	
Quality assessment rating	
Outcomes assessed	
Results /findings	
Meta-analysis performed	
Effect size, CI, P-value of meta-analysis of main outcomes	
Heterogeneity test and results	
GRADE	
Funding	
Comments	

Goel S, *et al. BMJ Open* 2024; 14:e080274. doi: 10.1136/bmjopen-2023-080274
