# Supplementary material for: Safety, efficacy and health impact of electronic nicotine delivery systems (ENDS): an umbrella review protocol
Source: BMJ Open. 2024 Jan 29;14(1):e080274. doi: 10.1136/bmjopen-2023-080274 (PMC10826537; doi:10.1136/bmjopen-2023-080274)
Supplement: Author's manuscript [file bmjopen-2023-080274.draft_revisions.pdf]

BMJ Open

BMJ Open is committed to open peer review. As part of this commitment we make the peer review history of every article we publish publicly available.

When an article is published we post the peer reviewers' comments and the authors' responses online. We also post the versions of the paper that were used during peer review. These are the versions that the peer review comments apply to.

The versions of the paper that follow are the versions that were submitted during the peer review process. They are not the versions of record or the final published versions. They should not be cited or distributed as the published version of this manuscript.

BMJ Open is an open access journal and the full, final, typeset and author-corrected version of record of the manuscript is available on our site with no access controls, subscription charges or pay-per-view fees (http://bmjopen.bmj.com).

If you have any questions on BMJ Open's open peer review process please email info.bmjopen@bmj.com

BMJ Open

Safety, efficacy and Health impact of Electronic Nicotine Delivery Systems (ENDS): An Umbrella Review protocol

Journal:	*BMJ Open*
Manuscript ID	bmjopen-2023-080274
Article Type:	Protocol
Date Submitted by the Author:	27-Sep-2023

Complete List of Authors:	Goel, Sonu; University of Limerick, School of Medicine, Faculty of Education & Health Sciences; Post Graduate Institute of Medical Education and Research<br>Shabil, Muhammed; Post Graduate Institute of Medical Education and Research<br>Kaur, Jagdish; World Health Organization Regional Office for South-East Asia<br>Chauhan, Anil; Post Graduate Institute of Medical Education and Research, Department of Telemedicine<br>Rinkoo, Arvind Vashishta; World Health Organization Regional Office for South-East Asia<br>Pradhan, Pranita; Post Graduate Institute of Medical Education and Research

Keywords:	Systematic Review, PUBLIC HEALTH, Health & safety < HEALTH SERVICES ADMINISTRATION & MANAGEMENT

SCHOLARONE™
Manuscripts

Safety, efficacy and Health impact of Electronic Nicotine Delivery Systems (ENDS): An Umbrella Review protocol

Sonu Goel[1,2,3*], Muhammed Shabil[2], Jagdish Kaur[4], Anil Chauhan[5], Arvind Vashishta Rinkoo[4], Pranita Pradhan[2]

Author affiliations

[1]School of Medicine, Faculty of Education & Health Sciences, University of Limerick, Ireland

[2]Post Graduate Institute of Medical Education and Research, Chandigarh-160012, India

[3]Faculty of Human and Health Sciences, Swansea University, United Kingdom

[4]Tobacco Free Initiative, World Health Organization Regional Office for South-East Asia, New Delhi, India

[5] Department of Telemedicine, Post Graduate Institute of Medical Education and Research, Chandigarh-160012, India

***Corresponding Author**

Dr. Sonu Goel

Adjunct Associate Clinical Professor

School of Medicine, Faculty of Education & Health Sciences

University of Limerick, Ireland

Email: sonugoel007@yahoo.co.in

Tel: (+91) 172 2755215, (+91) 172 2755220

ORCID: https://orcid.org/0000-0001-5231-7083

Word count: 1184

For peer review only

ABSTRACT

Background: Electronic nicotine delivery systems (ENDS), commonly known as e-cigarettes or vapes, have witnessed a rise in popularity, particularly among the youth. Although they were initially introduced as an alternative to traditional smoking, the design and function of ENDS vary. The potential health effects of ENDS, especially in comparison to traditional cigarettes, are a matter of ongoing debate. Given the increasing number of clinical studies and systematic reviews on this topic, there exists a demand for an umbrella review that offers a comprehensive assessment. The goal of this study is to perform an umbrella review of systematic reviews and meta-analyses (SRMAs) to assess the safety, efficacy, health implications, and potential gateway effect associated with ENDS.

Methods and analysis: This umbrella review will adhere to the Joanna Briggs Institute (JBI) framework and the PRISMA guidelines. A planned literature search will be executed across databases such as OVID, PubMed/MEDLINE, EMBASE, Cochrane Library, and Web of Science. The inclusion criteria are systematic reviews that discuss ENDS and e-liquids in the context of safety, efficacy, and health outcomes. The exclusion criteria include narrative reviews, non-systematic reviews, and studies not in English. Quality of the selected studies will be evaluated using the AMSTAR-2 scale. An overlap assessment will be done using the Corrected Covered Area (CCA), and data synthesis will be presented both narratively and in tabulated forms

Ethics and dissemination: Ethics approval is not required for this study as it does not involve the collection of original data. The results will be disseminated through peer-reviewed publication. The findings will offer crucial insights for stakeholders, policymakers, and the general public, underlining the health implications and the role of ENDS in tobacco cessation.

Keywords: Nicotine, Umbrella review, Tobacco, Protocol, vaping

For peer review only

Strengths and limitations of this study

- The umbrella review approach allows a thorough synthesis of existing reviews on ENDS, offering insights into safety, efficacy, and health implications.
- Adherence to the JBI framework and PRISMA guidelines ensures methodological rigor and transparent review reporting.
- The insights provided have practical relevance and applicability for stakeholders, policymakers, and the general public.
- Excluding non-English studies may introduce language bias, overlooking significant findings in other languages.
- Reliance on existing reviews means inherent gaps, limitations, or biases in them will affect this study's conclusions.

For peer review only - http://bmjopen.bmj.com/site/about/guidelines.xhtml

INTRODUCTION

Electronic cigarettes, commonly known as e-cigarettes or vapes, are devices designed to aerosolize a substance called "e-liquid" for inhalation (1-3). These devices, also known as electronic nicotine delivery systems (ENDS), were first created in 2003 with the intention of serving as a tool to help individuals quit smoking traditional cigarettes (4). ENDS function by utilizing a heating element to heat the e-liquid, producing a vapor that can be inhaled through a mouthpiece. During this process, new chemical compounds may be generated, some of which could pose health risks. E-cigarette devices come in various forms, ranging from older, lower-power models resembling traditional cigarettes (often called "cigalikes") to refillable pens, larger tank systems, and more recent innovations like compact devices using high-concentration nicotine salt pods and disposable options (3, 5). E-cigarettes have gained widespread popularity and are used by millions of people globally, with a notable prevalence among younger individuals (6, 7) .

The significantly reduced levels of harmful substances in ENDS compared to cigarettes have prompted researchers to explore their potential for assisting with smoking cessation (8, 9). However, concerns about the negative health impacts of second-hand aerosol exposure remain. The limited regulation of these products might also play a role in the expansion of the ENDS market, where tobacco companies have a notable presence. This could potentially lead to a resurgence of smoking habits, undermining years of anti-tobacco efforts in the Southeast Asian (SEA) Region. There has been a surge in clinical studies on ENDS, and the Cochrane Group published the first systematic review on ENDS in 2014 (10). In the recent years, there has been an increase in the publication of systematic reviews and meta-analyses (SRMAs) covering various aspects of ENDS to assess their effectiveness in aiding tobacco cessation (11-19).

The present circumstances necessitate mobilizing policymakers to address this issue in a region where a substantial burden of tobacco use is exacerbated by a significant population of susceptible young individuals and a limited well-established tobacco cessation resource. Because of the need for a comprehensive approach, an additional step in synthesizing existing SRMAs has been established in the form of umbrella reviews. Umbrella reviews are conducted consistently, enabling a comprehensive analysis by integrating existing systematic reviews and meta-analyses SRMAs. They swiftly assess abundant evidence, comparing prior systematic reviews and achieving coherence by subdividing complex issues into specific populations or interventions (20). The purpose of this umbrella review is to evaluate the impact of ENDS on health and its efficacy and safety in tobacco cessation.

Objectives

The aim of present study is to conduct an umbrella review of SRMAs to evaluate safety, efficacy, health outcomes, and gateway effect of ENDS.

Objective 1: To assess the effectiveness of ENDS as a tool for tobacco cessation by investigating quit rates among tobacco smokers using ENDS.

Objective 2: To identify and analyze adverse effects associated with the use of ENDS.

For peer review only - http://bmjopen.bmj.com/site/about/guidelines.xhtml

Objective 3: To evaluate the short-term and long-term health outcomes linked to the utilization of ENDS.

Objective 4: To explore the potential gateway effect of ENDS, particularly in relation to the initiation of tobacco smoking among individuals who were either never tobacco users or had previously quit smoking

METHODS

The method for conducting this umbrella review is based on the framework set forth by the Joanna Briggs Institute (JBI) (20). We will consistently follow the PRISMA guidelines throughout the entirety of our process. A checklist, derived from the Preferred Reporting Items for Systematic Review and Meta-Analysis Protocols (PRISMA-P), has been filled out and is available in **Supplementary Table 1** (21). Our umbrella review protocol will be registered in the PROSPERO. Any adjustments to our methodology will be documented and thoroughly explained in the final umbrella review report.

Inclusion and exclusion criteria

We will include systematic reviews in our evaluation, even if they form part of broader assessments. These broader assessments may cover topics such as the safety and efficacy of ENDS for tobacco smoking cessation or reduction, health-related outcomes associated with ENDS use in humans, and the potential initiation of tobacco smoking by never smokers or former tobacco users due to ENDS (referred to as the "Gateway effect").

The interventions include e-cigarettes, ENDS, and e-liquids. Notably, non-nicotine e-cigarettes and other pharmacological treatments, such as nicotine replacement therapy, are excluded.

For assessing efficacy, quitting rates of combustible tobacco smoking among those who are using ENDS will be considered. These rates can range from a period of 1 month to one year, based on data from primary studies. Reduction in the number of cigarettes smoked, reduction in the number of cigarette-smoking days, and proportion of participants achieving a 50% reduction in tobacco smoking during the follow-up period will be considered. The risk of continuing tobacco smoking in both the intervention and comparison groups will also be evaluated.

For safety considerations, we will assess any adverse events linked to e-cigarettes. This includes but not limited to incidents such as poisoning, explosions, and health issues due to malfunctioning ENDS, as well as allergic reactions to any contained chemicals. Health outcomes are categorized into short-term and long-term. Short-term refers to immediate outcomes, and long-term encompasses outcomes observed over a span of months to years. The health outcomes of interest are:

Incidence and risk of clinical disease endpoints such as coronary artery disease, myocardial infarction, congestive heart failure, stroke, other cardiovascular diseases, and any type of cancer. Development of risk factors and intermediate biological effects on health, including atherosclerosis, high blood pressure, lung damage, elevated glucose levels, and dyslipidemia. Incidence and risk of respiratory diseases, oral health complications, renal health concerns, neurological effects, optical health issues, impaired wound healing, olfactory issues,

For peer review only - http://bmjopen.bmj.com/site/about/guidelines.xhtml

endocrine problems, allergic reactions, and hematological outcomes. Pregnancy-related risks, neonatal effects, developmental and reproductive issues, and corresponding changes in clinical parameters. Mental health effects, and the impact on sleep patterns, quality, and duration. All the health outcomes will be evaluated based on proportion, risk, or mean difference of clinical parameters.

To evaluate the potential "gateway effect" of ENDS, the incidence and risk of initiation of combustible tobacco cigarette smoking in non-smokers or former smokers who use ENDS will be considered.

Our inclusion criteria will extend to systematic reviews that incorporate observational studies or randomized controlled trials (RCTs). Additionally, we will consider more recent primary studies that have not been previously incorporated into existing systematic reviews. These primary studies will be RCTs, cohort, case control, non-randomized clinical trials, cross sectional studies. We will exclude narrative reviews, non-systematic reviews, commentaries, and editorial articles. Additionally, studies not available in English language or published in non-peer-reviewed journals will be excluded. The specific criteria for the population, intervention, comparator, and outcome (PICO) are detailed in **Table 1**, providing a comprehensive framework that delineates the scope of our umbrella review.

Table 1: PICO

PICO	Inclusion criteria	Exclusion Criteria
**Population**	General population with or without cigarette smokers with > 12 years age	Animals<br>In-vitro<br>In-vivo
**Intervention**	E-cigarettes, Electronic Nicotine delivery systems, e-liquids	Nicotine replacement therapy<br>Non-nicotine e-cigarettes<br>Other pharmacological interventions
**Comparison**	**For safety and efficacy: -**<br>Placebo e-cigarette (without nicotine) or any comparator treatment or combination of treatments usually given for smoking cessation, e.g: nicotine replacement therapy, Cigarette smokers without any treatment, without e- cigarette<br><br>**For health outcomes: -**<br>Never smokers (no e-cigarette or combustible tobacco products ever)<br>Smoker populations- if no other comparator available for some outcomes<br><br>**For gateway effect: -**<br><br>Never smoke, never E-cigarette users	Dual users of e-cigarette and tobacco
**Outcome**	**Primary Outcomes:**<br>a) Tobacco smoking cessation, 50% reduction in cigarette consumption,	Economic outcomes<br>Environmental outcomes

For peer review only - http://bmjopen.bmj.com/site/about/guidelines.xhtml

	Adverse events<br><br>b) Clinical disease endpoints, such as myocardial infarction, coronary artery disease, congestive heart failure, stroke, other cardiovascular disease and cancer<br><br>c) Development of risk factors and intermediate biological effect of health outcomes like atherosclerosis, high blood pressure, lung damage, high glucose levels, dyslipidemia<br><br>d) Respiratory diseases Oral health, Renal health Neurological effects optical health, wound healing, olfactory, endocrine, allergic diseases and haematological outcomes<br><br>e) Effect on pregnancy, neonatal effects, Development and reproductive effects<br><br>f) Mental health, effects on Sleep pattern, quality, duration<br><br>g) Gateway effect (Ever smoking combustible tobacco cigarettes)<br>h) Nicotine dependency<br>i) Serious and non-serious adverse effects	
**Study type**	Systematic reviews and meta-analyses of RCTS and observational studies<br>Primary studies (observational studies and RCTs)	case reports, non-human studies
**Setting**	Any country	No exclusion
**Followup**	No restrictions	No exclusion
**Language**	English	Not available in English

Databases and searching

We will conduct a comprehensive systematic literature search across the various databases such as OVID, PubMed/MEDLINE, EMBASE, Cochrane Library, and Web of Science. The search strategy will be optimized to enhance accuracy and comprehensiveness, if necessary. The search will be conducted by an experienced medical librarian. A search strategy for PubMed is given in **Table 2**. Keywords and MeSH terms related to ENDS will be used in the search process. The search will employ a study design filter to identify systematic reviews,

For peer review only - http://bmjopen.bmj.com/site/about/guidelines.xhtml

whenever available, within each database. A language filter for the English will be applied. We will not impose any date limits on the search, ensuring that we capture relevant literature spanning various time frames. Reviewers will examine the citations of the included articles to identify additional relevant articles.

Table 2: Search strategy for PubMed

	**PubMed**
#1	("e-cig*"[Title/Abstract] OR "ecig*"[Title/Abstract] OR "e cig*"[Title/Abstract] OR "electronic cig*"[Title/Abstract] OR "electronic nicotine*"[Title/Abstract] OR vape[Title/Abstract] OR vapes[Title/Abstract] OR vaporizer[Title/Abstract] OR vapourizer[Title/Abstract] OR vaporiser[Title/Abstract] OR vapouriser[Title/Abstract] OR vaper[Title/Abstract] OR vapers[Title/Abstract] OR vaping[Title/Abstract] OR e-liquid[Title/Abstract] OR ENDS[Title/Abstract])
#2	#1 AND Filters :"systematic review", "Meta-analysis", Language: English

Screening and selection

The search results will be consolidated and de-duplicated using Covidence. Screening processes will be conducted using Covidence for the initial screening of titles and abstracts, followed by full-text screening. Two independent reviewers will be responsible for evaluating the titles, abstracts, and full texts of articles to determine their eligibility. Systematic reviews that align with the predefined PICO criteria will be included in the analysis. In cases of uncertainty or disagreement between the two reviewers, a third reviewer will assess the article to reach a consensus and make a final determination regarding its inclusion or exclusion.

Data extraction

Data extraction will be carried out by two different reviewers independently, and a pre-piloted and standardized data extraction form by JBI will be used. In cases where there are disagreements in data extraction, a third reviewer will be consulted to facilitate discussion and reach a consensus. Both quantitative and qualitative from each included study will be extracted. The extracted information will be displayed in a tabular format for clear and concise presentation accompanied by explanatory text. The quantitative compilation of results will include details such as the first author's name, publication year, study setting, the number of RCTs and observational studies encompassed in the systematic review, characteristics of the study participants, specifics of the interventions and comparators employed, and the outcomes assessed. This will also cover the total number of participants, effect size with their confidence intervals (CI), metrics and results for heterogeneity, results pertaining to publication bias and the tests utilized, and the type of quality assessment tool implemented along with its results. Additionally, values for the total pooled effects, Cochran Q statistic, Egger's test, and I^2 will be extracted.

Additionally, information regarding the funding sources of funding for systematic reviews and any potential conflicts of interest, especially concerning financial benefits related to the intervention, will also be extracted. A data extraction form template is given as **Supplementary Table 2.**

For peer review only - http://bmjopen.bmj.com/site/about/guidelines.xhtml

Quality assessment

The quality assessment of included SRMAs will be conducted by two reviewers independently using the AMSTAR-2 scale (22). A third reviewer will be consulted in case of difference of opinion. AMSTAR-2 consists of 16 domains, seven of which are classified as critical domains because of their substantial impact on confidence in the conclusions drawn from systematic reviews (22). These critical domains encompass a range of crucial aspects, including the registration of the review protocol, appropriateness of the search strategy, reason for excluding specific studies, risk of bias assessment of the included studies and its influence on systematic review's conclusions, method used for evidence synthesis, and consideration of publication bias. The systematic review's overall confidence level in its results will be categorized into four distinct levels: high, moderate, low, and very low (22).

Data synthesis

Prior to conducting the synthesis of findings, a sensitivity analysis will be carried out to assess the extent of overlap among primary studies across the systematic reviews. This analysis is essential as overlapping studies can lead to potential biases in the analysis. For this purpose, we have chosen the Corrected Covered Area (CCA) metric (23), a validated and widely used approach. To compute CCA, a citation matrix of primary studies will be created and this matrix will be included in our review. The CCA score will be categorized into different levels, including very high (>15), high (11-15), moderate (6-10), and slight (0-5) (24). This analysis will help us manage and account for potential overlap among the primary studies.

The synthesis of evidence will be presented in both narrative and tabular formats. We will provide a table detailing the specifics of each systematic review included in our analysis, encompassing information such as the intervention studied, the target population, outcomes assessed, comparator, the number of primary studies and participants involved, the search databases used along with their respective dates, and the effect estimates reported, such as risk ratios (RR), odds ratios (OR), hazard ratios (HR), mean difference (MD), Standardised mean difference (SMD) or similar metrics when available and their CIs, heterogeneity, publication bias, final findings, quality assessments, and a summary of the risk of bias identified in the primary studies included. A narrative approach will be used to summarize the evidence for each outcome, and we will also employ tabular formats where applicable to enhance clarity. Weightage will be given to the results of highest rated systematic review by AMSTAR-2 where the reviews show a higher level of overlap. In instances where there are discrepancies between the results of recent and high-quality systematic reviews, we will conduct a re-analysis of the primary data to arrive at a conclusion.

If newer primary studies necessitate data synthesis, a meta-analysis will be performed if more than three studies are available for each outcome. Meta-analysis will be conducted by pooling the effect size from each study using a random-effects model. The R software will be employed for the meta-analysis. Subgroup analyses will be undertaken based on variables such as participants, outcomes, or any other relevant factors that might contribute to heterogeneity. The I^2 statistic will be used to assess heterogeneity. A p-value of less than 0.05 will be deemed statistically significant. Publication bias will be assessed through visual inspection of funnel plot symmetry and the Egger's test. Sensitivity analysis will be performed to determine the effect of each study on the overall result.

For peer review only - http://bmjopen.bmj.com/site/about/guidelines.xhtml

The quality of evidence will be determined using GRADE criteria (Grading of Recommendations, Assessment, Development, and Evaluations). Garding will be performed by considering 5 domains including risk of bias in the individual studies, inconsistency, indirectness, imprecision, and publication bias for each outcome (25). We will grade the strength of evidence (very low, low, moderate and high).

Ethics and dissemination: Ethics approval is not required for this study as it does not involve the collection of original data. The results will be disseminated through peer-reviewed publication. The findings will offer crucial insights for stakeholders, policymakers, and the general public, underlining the health implications and the role of ENDS in tobacco cessation.

Ethics approval and consent to participate: No ethical approval required

Patient consent for publication: Not required

Funding: The review is funded by World Health Organisation

Conflict of interest: The authors declare that they have no competing interests

Author Contributions.

Guarantor: SG. SG, JK, and AVR conceptualized the topic. AC and MS analyzed and finalized the methods. MS, AC drafted the manuscript. PP developed the search strategy. SG, JK, AVR, and AC reviewed, edited and proofread the final draft. SG, MS, JK, AVR, AC and PP finalized and approved the manuscript.

Patient and Public Involvement statement: Patients or the public were not involved in the design, or conduct, or reporting, or dissemination plans of our research

REFERENCES

1. DHHS U. E-cigarette use among youth and young adults A report of the Surgeon General. Atlanta, GA: US DHHS, CDC. National Center for Chronic Disease Prevention and Health Promotion, Office on Smoking and Health. 2016.
2. de Jong WH, Panagiotakos DB, Proykova A, Samaras T, Clemens MW, De Jong D, et al. Scientific Committee on Health, Environmental and Emerging Risks (SCHEER): final opinion on the safety of breast implants in relation to anaplastic large cell lymphoma. 2021.
3. National Academies of Sciences E, Medicine. Public health consequences of e-cigarettes. 2018.
4. Boseley S. Hon Lik invented the e-cigarette to quit smoking–but now he's a dual user. The Guardian. 2015;19.
5. Hajek P, Przulj D, Phillips A, Anderson R, McRobbie H. Nicotine delivery to users from cigarettes and from different types of e-cigarettes. Psychopharmacology. 2017;234:773-9.
6. MARTINS BNFL. FREQUÊNCIA GLOBAL E PERFIL EPIDEMIOLÓGICO DOS USUÁRIOS DE CIGARRO ELETRÔNICO: UMA REVISÃO SISTEMÁTICA.
7. Yoong SL, Hall A, Leonard A, McCrabb S, Wiggers J, d'Espaignet ET, et al. Prevalence of electronic nicotine delivery systems and electronic non-nicotine delivery systems in children and adolescents: a systematic review and meta-analysis. The Lancet Public Health. 2021;6(9):e661-e73.
8. Eaton DL, Alberg AJ, Goniewicz M, Leventhal A, Manautou JE, McGrath-Morrow S, et al. Committee on the Review of the Health Effects of Electronic Nicotine Delivery Systems. Public

For peer review only - http://bmjopen.bmj.com/site/about/guidelines.xhtml

health consequences of e-cigarettes Consensus study report for the US Food and Drug Administration Washington (DC): US National Academies of Science, Medicine and Engineering. 2018.
9. Goniewicz ML, Smith DM, Edwards KC, Blount BC, Caldwell KL, Feng J, et al. Comparison of nicotine and toxicant exposure in users of electronic cigarettes and combustible cigarettes. JAMA network open. 2018;1(8):e185937-e.
10. McRobbie H, Bullen C, Hartmann-Boyce J, Hajek P. Electronic cigarettes for smoking cessation and reduction. Cochrane database of systematic reviews. 2014(12).
11. Bravo-Gutiérrez OA, Falfán-Valencia R, Ramírez-Venegas A, Sansores RH, Ponciano-Rodríguez G, Pérez-Rubio G. Lung damage caused by heated tobacco products and electronic nicotine delivery systems: a systematic review. International journal of environmental research and public health. 2021;18(8):4079.
12. Tzortzi A, Kapetanstrataki M, Evangelopoulou V, Behrakis P. A systematic literature review of e-cigarette-related illness and injury: not just for the respirologist. International journal of environmental research and public health. 2020;17(7):2248.
13. Yang I, Sandeep S, Rodriguez J. The oral health impact of electronic cigarette use: a systematic review. Critical reviews in toxicology. 2020;50(2):97-127.
14. Li X, Zhang Y, Zhang R, Chen F, Shao L, Zhang L. Association between e-cigarettes and asthma in adolescents: a systematic review and meta-analysis. American Journal of Preventive Medicine. 2022;62(6):953-60.
15. Jonas AM, Raj R. Vaping-related acute parenchymal lung injury: a systematic review. Chest. 2020;158(4):1555-65.
16. Kalkhoran S, Glantz SA. E-cigarettes and smoking cessation in real-world and clinical settings: a systematic review and meta-analysis. The Lancet Respiratory Medicine. 2016;4(2):116-28.
17. Skotsimara G, Antonopoulos AS, Oikonomou E, Siasos G, Ioakeimidis N, Tsalamandris S, et al. Cardiovascular effects of electronic cigarettes: a systematic review and meta-analysis. European journal of preventive cardiology. 2019;26(11):1219-28.
18. Kennedy CD, van Schalkwyk MC, McKee M, Pisinger C. The cardiovascular effects of electronic cigarettes: a systematic review of experimental studies. Preventive medicine. 2019;127:105770.
19. O'Brien D, Long J, Quigley J, Lee C, McCarthy A, Kavanagh P. Association between electronic cigarette use and tobacco cigarette smoking initiation in adolescents: a systematic review and meta-analysis. BMC Public Health. 2021;21(1):1-10.
20. Aromataris E, Fernandez R, Godfrey CM, Holly C, Khalil H, Tungpunkom P. Summarizing systematic reviews: methodological development, conduct and reporting of an umbrella review approach. JBI Evidence Implementation. 2015;13(3):132-40.
21. Shamseer L, Moher D, Clarke M, Ghersi D, Liberati A, Petticrew M, et al. Preferred reporting items for systematic review and meta-analysis protocols (PRISMA-P) 2015: elaboration and explanation. Bmj. 2015;349.
22. Shea BJ, Reeves BC, Wells G, Thuku M, Hamel C, Moran J, et al. AMSTAR 2: a critical appraisal tool for systematic reviews that include randomised or non-randomised studies of healthcare interventions, or both. bmj. 2017;358.
23. Pieper D, Antoine S-L, Mathes T, Neugebauer EA, Eikermann M. Systematic review finds overlapping reviews were not mentioned in every other overview. Journal of clinical epidemiology. 2014;67(4):368-75.
24. Hennessy EA, Johnson BT. Examining overlap of included studies in meta-reviews: Guidance for using the corrected covered area index. Research synthesis methods. 2020;11(1):134-45.
25. Langendam MW, Akl EA, Dahm P, Glasziou P, Guyatt G, Schünemann HJ. Assessing and presenting summaries of evidence in Cochrane Reviews. Systematic reviews. 2013;2(1):1-9.

Tables

For peer review only - http://bmjopen.bmj.com/site/about/guidelines.xhtml

Table 1: PICO

Table 2: Search strategy for PubMed

Supplementary table 1: PRISMA-P

Supplementary table 2: Pre-piloted data extraction form

For peer review only

For peer review only - http://bmjopen.bmj.com/site/about/guidelines.xhtml

Supplementary materials

Supplementary table 1: PRISMA-P

Section and topic	Item No	Checklist item	Location
**ADMINISTRATIVE INFORMATION**			
Title:			
Identification	1a	Identify the report as a protocol of a systematic review	Page 1 (Umbrella review)
Update	1b	If the protocol is for an update of a previous systematic review, identify as such	N/A
Registration	2	If registered, provide the name of the registry (such as PROSPERO) and registration number	Page 4*
Authors:			
Contact	3a	Provide name, institutional affiliation, e-mail address of all protocol authors; provide physical mailing address of corresponding author	Page 1
Contributions	3b	Describe contributions of protocol authors and identify the guarantor of the review	Page 7
Amendments	4	If the protocol represents an amendment of a previously completed or published protocol, identify as such and list changes; otherwise, state plan for documenting important protocol amendments	Page 4
Support:			
Sources	5a	Indicate sources of financial or other support for the review	Page 7
Sponsor	5b	Provide name for the review funder and/or sponsor	Page 7
Role of sponsor or funder	5c	Describe roles of funder(s), sponsor(s), and/or institution(s), if any, in developing the protocol	Page 7
**INTRODUCTION**			
Rationale	6	Describe the rationale for the review in the context of what is already known	Page 3
Objectives	7	Provide an explicit statement of the question(s) the review will address with reference to participants, interventions, comparators, and outcomes (PICO)	Page 3,4
**METHODS**			
Eligibility criteria	8	Specify the study characteristics (such as PICO, study design, setting, time frame) and report characteristics (such as years considered, language, publication status) to be used as criteria for eligibility for the review	Page 4,5, table 1
Information sources	9	Describe all intended information sources (such as electronic databases, contact with study authors, trial registers or other grey literature sources) with planned dates of coverage	Page 5
Search strategy	10	Present draft of search strategy to be used for at least one electronic database, including planned limits, such that it could be repeated	Table 2
Study records:			
Data management	11a	Describe the mechanism(s) that will be used to manage records and data throughout the review	Page 7

For peer review only - http://bmjopen.bmj.com/site/about/guidelines.xhtml

Selection process	11b	State the process that will be used for selecting studies (such as two independent reviewers) through each phase of the review (that is, screening, eligibility and inclusion in meta-analysis)	Page 7
Data collection process	11c	Describe planned method of extracting data from reports (such as piloting forms, done independently, in duplicate), any processes for obtaining and confirming data from investigators	Page 7 Supplementary table 2
Data items	12	List and define all variables for which data will be sought (such as PICO items, funding sources), any pre-planned data assumptions and simplifications	Page 7 Supplementary table 2
Outcomes and prioritization	13	List and define all outcomes for which data will be sought, including prioritization of main and additional outcomes, with rationale	Page 7
Risk of bias in individual studies	14	Describe anticipated methods for assessing risk of bias of individual studies, including whether this will be done at the outcome or study level, or both; state how this information will be used in data synthesis	Page 8
Data synthesis	15a	Describe criteria under which study data will be quantitatively synthesized	Page 8
	15b	If data are appropriate for quantitative synthesis, describe planned summary measures, methods of handling data and methods of combining data from studies, including any planned exploration of consistency (such as I^2, Kendall's τ)	Page 78
	15c	Describe any proposed additional analyses (such as sensitivity or subgroup analyses, meta-regression)	Page 78
	15d	If quantitative synthesis is not appropriate, describe the type of summary planned	Page 8
Meta-bias(es)	16	Specify any planned assessment of meta-bias(es) (such as publication bias across studies, selective reporting within studies)	Page 8
Confidence in cumulative evidence	17	Describe how the strength of the body of evidence will be assessed (such as GRADE)	Page 9

*Pending

Supplementary table 2: pre-piloted data extraction form

Doi	
Author and Year	
Objectives	
Details of sources searched	
Type of included studies	

For peer review only - http://bmjopen.bmj.com/site/about/guidelines.xhtml

No of included studies	
Country of included studies	
Participants (characteristics/total number)	
Description of Intervention	
Range (years) of included studies	
Quality assessment tool used	
Quality assessment rating	
Outcomes assessed	
Results /findings	
Meta-analysis performed	
Effect size, CI, P-value of meta-analysis of main outcomes	
Heterogeneity test and results	
GRADE	
Funding	
Comments	

For peer review only

For peer review only - http://bmjopen.bmj.com/site/about/guidelines.xhtml

PRISMA-P checklist

Section and topic	Item No	Checklist item	Location
**ADMINISTRATIVE INFORMATION**			
Title:			
Identification	1a	Identify the report as a protocol of a systematic review	Page 1 (Umbrella review)
Update	1b	If the protocol is for an update of a previous systematic review, identify as such	N/A
Registration	2	If registered, provide the name of the registry (such as PROSPERO) and registration number	Page 4*
Authors:			
Contact	3a	Provide name, institutional affiliation, e-mail address of all protocol authors; provide physical mailing address of corresponding author	Page 1
Contributions	3b	Describe contributions of protocol authors and identify the guarantor of the review	Page 7
Amendments	4	If the protocol represents an amendment of a previously completed or published protocol, identify as such and list changes; otherwise, state plan for documenting important protocol amendments	Page 4
Support:			
Sources	5a	Indicate sources of financial or other support for the review	Page 7
Sponsor	5b	Provide name for the review funder and/or sponsor	Page 7
Role of sponsor or funder	5c	Describe roles of funder(s), sponsor(s), and/or institution(s), if any, in developing the protocol	Page 7
**INTRODUCTION**			
Rationale	6	Describe the rationale for the review in the context of what is already known	Page 3
Objectives	7	Provide an explicit statement of the question(s) the review will address with reference to participants, interventions, comparators, and outcomes (PICO)	Page 3,4
**METHODS**			
Eligibility criteria	8	Specify the study characteristics (such as PICO, study design, setting, time frame) and report characteristics (such as years considered, language, publication status) to be used as criteria for eligibility for the review	Page 4,5, table 1
Information sources	9	Describe all intended information sources (such as electronic databases, contact with study authors, trial registers or other grey literature sources) with planned dates of coverage	Page 5
Search strategy	10	Present draft of search strategy to be used for at least one electronic database, including planned limits, such that it could be repeated	Table 2
Study records:			
Data management	11a	Describe the mechanism(s) that will be used to manage records and data throughout the review	Page 7

For peer review only

Selection process	11b	State the process that will be used for selecting studies (such as two independent reviewers) through each phase of the review (that is, screening, eligibility and inclusion in meta-analysis)	Page 7
Data collection process	11c	Describe planned method of extracting data from reports (such as piloting forms, done independently, in duplicate), any processes for obtaining and confirming data from investigators	Page 7 Supplementary table 2
Data items	12	List and define all variables for which data will be sought (such as PICO items, funding sources), any pre-planned data assumptions and simplifications	Page 7 Supplementary table 2
Outcomes and prioritization	13	List and define all outcomes for which data will be sought, including prioritization of main and additional outcomes, with rationale	Page 7
Risk of bias in individual studies	14	Describe anticipated methods for assessing risk of bias of individual studies, including whether this will be done at the outcome or study level, or both; state how this information will be used in data synthesis	Page 8
Data synthesis	15a	Describe criteria under which study data will be quantitatively synthesized	Page 8
	15b	If data are appropriate for quantitative synthesis, describe planned summary measures, methods of handling data and methods of combining data from studies, including any planned exploration of consistency (such as I^2, Kendall's τ)	Page 78
	15c	Describe any proposed additional analyses (such as sensitivity or subgroup analyses, meta-regression)	Page 78
	15d	If quantitative synthesis is not appropriate, describe the type of summary planned	Page 8
Meta-bias(es)	16	Specify any planned assessment of meta-bias(es) (such as publication bias across studies, selective reporting within studies)	Page 8
Confidence in cumulative evidence	17	Describe how the strength of the body of evidence will be assessed (such as GRADE)	Page 9

For peer review only

Safety, efficacy and Health impact of Electronic Nicotine Delivery Systems (ENDS): An Umbrella Review protocol

Journal:	*BMJ Open*
Manuscript ID	bmjopen-2023-080274.R1
Article Type:	Protocol
Date Submitted by the Author:	03-Dec-2023
Complete List of Authors:	Goel, Sonu; University of Limerick; Post Graduate Institute of Medical Education and Research, Department of Community Medicine and School of Public Health<br>Shabil, Muhammed; Post Graduate Institute of Medical Education and Research<br>Kaur, Jagdish; World Health Organization Regional Office for South-East Asia<br>Chauhan, Anil; Post Graduate Institute of Medical Education and Research, Department of Telemedicine<br>Rinkoo, Arvind Vashishta; World Health Organization Regional Office for South-East Asia
**Primary Subject Heading**:	Public health
Secondary Subject Heading:	Evidence based practice, Health policy
Keywords:	Systematic Review, PUBLIC HEALTH, Health & safety < HEALTH SERVICES ADMINISTRATION & MANAGEMENT

SCHOLARONE™
Manuscripts

Safety, efficacy and Health impact of Electronic Nicotine Delivery Systems (ENDS): An Umbrella Review protocol

Sonu Goel[1,2,3*], Muhammed Shabil[2], Jagdish Kaur[4], Anil Chauhan[5], Arvind Vashishta Rinkoo[4],

Author affiliations

[1]School of Medicine, Faculty of Education & Health Sciences, University of Limerick, Ireland

[2]Post Graduate Institute of Medical Education and Research, Chandigarh-160012, India

[3]Faculty of Human and Health Sciences, Swansea University, United Kingdom

[4]Tobacco Free Initiative, World Health Organization Regional Office for South-East Asia, New Delhi, India

[5] Department of Telemedicine, Post Graduate Institute of Medical Education and Research, Chandigarh-160012, India

***Corresponding Author**

Dr. Sonu Goel

Adjunct Associate Clinical Professor

School of Medicine, Faculty of Education & Health Sciences

University of Limerick, Ireland

Email: sonu.goel@ul.ie

Tel: (+91) 172 2755215, (+91) 172 2755220

ORCID: https://orcid.org/0000-0001-5231-7083

Word count: 1316

For peer review only - http://bmjopen.bmj.com/site/about/guidelines.xhtml

ABSTRACT

Background: Electronic nicotine delivery systems (ENDS), commonly known as e-cigarettes or vapes, have witnessed a rise in popularity, particularly among the youth. Although they were initially introduced as an alternative to traditional smoking, the design and function of ENDS vary. The potential health effects of ENDS, especially in comparison to traditional cigarettes, are a matter of ongoing debate. Given the increasing number of clinical studies and systematic reviews on this topic, there exists a demand for an umbrella review that offers a comprehensive assessment. The goal of this study is to perform an umbrella review of systematic reviews and meta-analyses (SRMAs) to assess the safety, efficacy, health implications, and potential gateway effect associated with ENDS.

Methods and analysis: This umbrella review will adhere to the Joanna Briggs Institute (JBI) framework and the PRISMA guidelines. A planned literature search will be executed across databases such as OVID, PubMed/MEDLINE, EMBASE, Cochrane Library, and Web of Science. The inclusion criteria are systematic reviews that discuss ENDS and e-liquids in the context of safety, efficacy, and health outcomes. The exclusion criteria include narrative reviews, non-systematic reviews, and studies not in English. Quality of the selected studies will be evaluated using the AMSTAR-2 scale. An overlap assessment will be done using the Corrected Covered Area (CCA), and data synthesis will be presented both narratively and in tabulated forms

Ethics and dissemination: Ethics approval is not required for this study as it does not involve the collection of original data. The results will be disseminated through peer-reviewed publication. The findings will offer crucial insights for stakeholders, policymakers, and the general public, underlining the health implications and the role of ENDS in tobacco cessation.

Keywords: Nicotine, Umbrella review, Tobacco, Protocol, vaping

Strengths and limitations of this study

- The umbrella review approach allows a thorough synthesis of existing reviews on ENDS, offering insights into safety, efficacy, and health implications.
- Adherence to the JBI framework and PRISMA guidelines ensures methodological rigor and transparent review reporting.
- The insights provided have practical relevance and applicability for stakeholders, policymakers, and the general public.
- Excluding non-English studies may introduce language bias, overlooking significant findings in other languages.
- Reliance on existing reviews means inherent gaps, limitations, or biases in them will affect this study's conclusions.

For peer review only - http://bmjopen.bmj.com/site/about/guidelines.xhtml

INTRODUCTION

Electronic cigarettes, commonly known as e-cigarettes or vapes, are devices designed to aerosolize a substance called "e-liquid" for inhalation (1-3). These devices, also known as electronic nicotine delivery systems (ENDS), were first created in 2003 with the intention of serving as a tool to help individuals quit smoking traditional cigarettes (4). ENDS function by utilizing a heating element to heat the e-liquid, producing a vapor that can be inhaled through a mouthpiece. During this process, new chemical compounds may be generated, some of which could pose health risks. E-cigarette devices come in various forms, ranging from older, lower-power models resembling traditional cigarettes (often called "cigalikes") to refillable pens, larger tank systems, and more recent innovations like compact devices using high-concentration nicotine salt pods and disposable options (3, 5). E-cigarettes have gained widespread popularity and are used by millions of people globally, with a notable prevalence among younger individuals (6, 7) .

The significantly reduced levels of harmful substances in ENDS compared to cigarettes have prompted researchers to explore their potential for assisting with smoking cessation (8, 9). However, concerns about the negative health impacts of second-hand aerosol exposure remain. The limited regulation of these products might also play a role in the expansion of the ENDS market, where tobacco companies have a notable presence. This could potentially lead to a resurgence of smoking habits, undermining years of anti-tobacco efforts in the Southeast Asian (SEA) Region. There has been a surge in clinical studies on ENDS, and the Cochrane Group published the first systematic review on ENDS in 2014 (10). In the recent years, there has been an increase in the publication of systematic reviews and meta-analyses (SRMAs) covering various aspects of ENDS to assess their effectiveness in aiding tobacco cessation (11-19).

The present circumstances necessitate mobilizing policymakers to address this issue in a region where a substantial burden of tobacco use is exacerbated by a significant population of susceptible young individuals and a limited well-established tobacco cessation resource. Because of the need for a comprehensive approach, an additional step in synthesizing existing SRMAs has been established in the form of umbrella reviews. Umbrella reviews are conducted consistently, enabling a comprehensive analysis by integrating existing systematic reviews and meta-analyses SRMAs. They swiftly assess abundant evidence, comparing prior systematic reviews and achieving coherence by subdividing complex issues into specific populations or interventions (20). The purpose of this umbrella review is to evaluate the impact of ENDS on health and its efficacy and safety in tobacco cessation.

Objectives

The aim of present study is to conduct three umbrella reviews of SRMAs to evaluate safety, efficacy, health outcomes, and gateway effect of ENDS. Three umbrella reviews will be conducted.

For peer review only - http://bmjopen.bmj.com/site/about/guidelines.xhtml

Umbrella review 1:

Objective 1: To assess the effectiveness of ENDS as a tool for tobacco cessation by investigating quit rates among tobacco smokers using ENDS.

Objective 2: To identify and analyze adverse effects associated with the use of ENDS.

Umbrella review 2:

Objective: To evaluate the short-term and long-term health outcomes linked to the utilization of ENDS.

Umbrella review 3:

Objective: To explore the potential gateway effect of ENDS, particularly in relation to the initiation of tobacco smoking among individuals who were either never tobacco users or had previously quit smoking

METHODS

Patient and Public Involvement statement: Patients or the public were not involved in the design, or conduct, or reporting, or dissemination plans of our research

The method for conducting this umbrella review is based on the framework set forth by the Joanna Briggs Institute (JBI) (20). We will consistently follow the PRISMA guidelines throughout the entirety of our process. A checklist, derived from the Preferred Reporting Items for Systematic Review and Meta-Analysis Protocols (PRISMA-P), has been filled out and is available in **Supplementary Table 1** (21). Our umbrella review protocol will be registered in the PROSPERO. Any adjustments to our methodology will be documented and thoroughly explained in the final umbrella review report. The study will commence on October 1, 2023, and continue until July 2024.

Inclusion and exclusion criteria

We will include systematic reviews in our evaluation, even if they form part of broader assessments. These broader assessments may cover topics such as the safety and efficacy of ENDS for tobacco smoking cessation or reduction, health-related outcomes associated with ENDS use in humans, and the potential initiation of tobacco smoking by never smokers or former tobacco users due to ENDS (referred to as the "Gateway effect").

The interventions include e-cigarettes, ENDS, and e-liquids. Notably, non-nicotine e-cigarettes and other pharmacological treatments, such as nicotine replacement therapy, are excluded.

For assessing efficacy, quitting rates of combustible tobacco smoking among those who are using ENDS will be considered. These rates can range from a period of 1 month to one year,

For peer review only - http://bmjopen.bmj.com/site/about/guidelines.xhtml

based on data from primary studies. Reduction in the number of cigarettes smoked, reduction in the number of cigarette-smoking days, and proportion of participants achieving a 50% reduction in tobacco smoking during the follow-up period will be considered. The risk of continuing tobacco smoking in both the intervention and comparison groups will also be evaluated.

For safety considerations, we will assess any adverse events linked to e-cigarettes. This includes but not limited to incidents such as poisoning, explosions, and health issues due to malfunctioning ENDS, as well as allergic reactions to any contained chemicals. Health outcomes are categorized into short-term and long-term. Short-term refers to immediate outcomes, and long-term encompasses outcomes observed over a span of months to years. The health outcomes of interest are:

Incidence and risk of clinical disease endpoints such as coronary artery disease, myocardial infarction, congestive heart failure, stroke, other cardiovascular diseases, and any type of cancer. Development of risk factors and intermediate biological effects on health, including atherosclerosis, high blood pressure, lung damage, elevated glucose levels, and dyslipidemia. Incidence and risk of respiratory diseases, oral health complications, renal health concerns, neurological effects, optical health issues, impaired wound healing, olfactory issues, endocrine problems, allergic reactions, and hematological outcomes. Pregnancy-related risks, neonatal effects, developmental and reproductive issues, and corresponding changes in clinical parameters. Mental health effects, and the impact on sleep patterns, quality, and duration. All the health outcomes will be evaluated based on proportion, risk, or mean difference of clinical parameters.

To evaluate the potential "gateway effect" of ENDS, the incidence and risk of initiation of combustible tobacco cigarette smoking in non-smokers or former smokers who use ENDS will be considered.

Our inclusion criteria will extend to systematic reviews that incorporate observational studies or randomized controlled trials (RCTs). Additionally, we will consider more recent primary studies that have not been previously incorporated into existing systematic reviews. These primary studies will be RCTs, cohort, case control, non-randomized clinical trials, cross sectional studies. We will exclude narrative reviews, non-systematic reviews, commentaries, and editorial articles. Additionally, studies not available in English language or published in non-peer-reviewed journals will be excluded. The specific criteria for the population, intervention, comparator, and outcome (PICO) are detailed in **Table 1**, providing a comprehensive framework that delineates the scope of our umbrella review.

Table 1: PICO

**PICO**	**Inclusion criteria**	**Exclusion Criteria**
**Population**	General population with or without cigarette smokers with > 12 years age	Animals<br>In-vitro<br>In-vivo

For peer review only - http://bmjopen.bmj.com/site/about/guidelines.xhtml

**Intervention**	E-cigarettes, Electronic Nicotine delivery systems, e-liquids	Nicotine replacement therapy<br>Non-nicotine e-cigarettes<br>Other pharmacological interventions
**Comparison**	**For safety and efficacy: -**<br>Placebo e-cigarette (without nicotine) or any comparator treatment or combination of treatments usually given for smoking cessation, e.g: nicotine replacement therapy, Cigarette smokers without any treatment, without e-cigarette<br><br>**For health outcomes: -**<br>Never smokers (no e-cigarette or combustible tobacco products ever)<br>Smoker populations- if no other comparator available for some outcomes<br><br>**For gateway effect: -**<br><br>Never smoke, never E-cigarette users	Dual users of e-cigarette and tobacco
**Outcome**	**Primary Outcomes:**<br>a) Tobacco smoking cessation, 50% reduction in cigarette consumption, Adverse events<br><br>b) Clinical disease endpoints, such as myocardial infarction, coronary artery disease, congestive heart failure, stroke, other cardiovascular disease and cancer<br><br>c) Development of risk factors and intermediate biological effect of health outcomes like atherosclerosis, high blood pressure, lung damage, high glucose levels, dyslipidemia<br><br>d) Respiratory diseases Oral health, Renal health Neurological effects optical health, wound healing, olfactory, endocrine, allergic diseases and haematological outcomes	Economic outcomes<br>Environmental outcomes

For peer review only

	e) Effect on pregnancy, neonatal effects, Development and reproductive effects<br>f) Mental health, effects on Sleep pattern, quality, duration<br>g) Gateway effect (Ever smoking combustible tobacco cigarettes)<br>h) Nicotine dependency<br>i) Serious and non-serious adverse effects	
**Study type**	Systematic reviews and meta-analyses of RCTS and observational studies<br>Primary studies (observational studies and RCTs)	case reports, non-human studies
**Setting**	Any country	No exclusion
**Followup**	No restrictions	No exclusion
**Language**	English	Not available in English

Databases and searching

We will conduct a comprehensive systematic literature search across the various databases such as OVID, PubMed/MEDLINE, EMBASE, Cochrane Library, CINAHL, and Web of Science. The search strategy will be optimized to enhance accuracy and comprehensiveness, if necessary. The search will be conducted by an experienced medical librarian. A search strategy for PubMed is given in **Table 2**. Keywords and MeSH terms related to ENDS will be used in the search process. The search will employ a study design filter to identify systematic reviews, whenever available, within each database. A language filter for the English will be applied. We will not impose any date limits on the search, ensuring that we capture relevant literature spanning various time frames. Reviewers will examine the citations of the included articles to identify additional relevant articles.

Table 2: Search strategy for PubMed

**Search**	**Query**	**Results**

For peer review only

#4	Search: **((((("Electronic Nicotine Delivery Systems"[Mesh]) OR ("Vaping"[Mesh])) OR (("e-cig*" OR "ecig*" OR "e cig*" OR "electronic cig*" OR "electronic nicotine*" OR vape OR vapes OR vaporizer OR vapourizer OR vaporiser OR vapouriser OR vaper OR vapers OR vaping OR e-liquid OR ENDS))) OR ((E Cigarettes) OR (E-Cigarette) OR (E Cigarette) OR (Electronic Cigarette) OR (Cigarette, Electronic) OR (Cigarettes, Electronic))) AND ((((("Adolescent"[Mesh]) OR ("Adult"[Mesh] OR "Young Adult"[Mesh])) OR (young people)) OR (middle aged)) OR (older adult)) OR (older people))) AND ((((systematic review) OR (systematic reviews)) OR (meta analysis)) OR (network meta analysis))**	1,564
#3	Search: **(((systematic review) OR (systematic reviews)) OR (meta analysis)) OR (network meta analysis)** Sort by: **Most Recent**	468,238
#2	Search: **((((("Adolescent"[Mesh]) OR ("Adult"[Mesh] OR "Young Adult"[Mesh])) OR (young people)) OR (middle aged)) OR (older adult)) OR (older people)**	9,046,337
#1	Search: **((("Electronic Nicotine Delivery Systems"[Mesh]) OR ("Vaping"[Mesh])) OR (("e-cig*" OR "ecig*" OR "e cig*" OR "electronic cig*" OR "electronic nicotine*" OR vape OR vapes OR vaporizer OR vapourizer OR vaporiser OR vapouriser OR vaper OR vapers OR vaping OR e-liquid OR ENDS))) OR ((E Cigarettes) OR (E-Cigarette) OR (E Cigarette) OR (Electronic Cigarette) OR (Cigarette, Electronic) OR (Cigarettes, Electronic))**	367,626

Screening and selection

The search results will be consolidated and de-duplicated using Covidence. Screening processes will be conducted using Covidence for the initial screening of titles and abstracts, followed by full-text screening. Two independent reviewers will be responsible for evaluating the titles, abstracts, and full texts of articles to determine their eligibility. Systematic reviews that align with the predefined PICO criteria will be included in the analysis. In cases of uncertainty or disagreement between the two reviewers, a third reviewer will assess the article to reach a consensus and make a final determination regarding its inclusion or exclusion.

Data extraction

Data extraction will be carried out by two different reviewers independently, and a pre-piloted and standardized data extraction form by JBI will be used. In cases where there are disagreements in data extraction, a third reviewer will be consulted to facilitate discussion and reach a consensus. Both quantitative and qualitative from each included study will be extracted. The extracted information will be displayed in a tabular format for clear and concise presentation accompanied by explanatory text. The quantitative compilation of results will include details such as the first author's name, publication year, study setting, the number of RCTs and observational studies encompassed in the systematic review, characteristics of the study participants, specifics of the interventions and comparators employed, and the outcomes assessed. This will also cover the total number of participants, effect size with their confidence intervals (CI), metrics and results for heterogeneity, results pertaining to publication bias and the tests utilized, and the type of quality assessment tool implemented along with its results. Additionally, values for the total pooled effects, Cochran Q statistic, Egger's test, and I^2 will be extracted.

For peer review only - http://bmjopen.bmj.com/site/about/guidelines.xhtml

Additionally, information regarding the funding sources of funding for systematic reviews and any potential conflicts of interest, especially concerning financial benefits related to the intervention, will also be extracted. A data extraction form template is given as **Supplementary Table 2.**

Quality assessment

The quality assessment of included SRMAs will be conducted by two reviewers independently using the AMSTAR-2 scale (22). A third reviewer will be consulted in case of difference of opinion. AMSTAR-2 consists of 16 domains, seven of which are classified as critical domains because of their substantial impact on confidence in the conclusions drawn from systematic reviews (22). These critical domains encompass a range of crucial aspects, including the registration of the review protocol, appropriateness of the search strategy, reason for excluding specific studies, risk of bias assessment of the included studies and its influence on systematic review's conclusions, method used for evidence synthesis, and consideration of publication bias. The systematic review's overall confidence level in its results will be categorized into four distinct levels: high, moderate, low, and very low (22).

Data synthesis

Prior to conducting the synthesis of findings, a sensitivity analysis will be carried out to assess the extent of overlap among primary studies across the systematic reviews. This analysis is essential as overlapping studies can lead to potential biases in the analysis. For this purpose, we have chosen the Corrected Covered Area (CCA) metric (23), a validated and widely used approach. To compute CCA, a citation matrix of primary studies will be created and this matrix will be included in our review. The CCA score will be categorized into different levels, including very high (>15), high (11-15), moderate (6-10), and slight (0-5) (24). This analysis will help us manage and account for potential overlap among the primary studies.

The synthesis of evidence will be presented in both narrative and tabular formats. We will provide a table detailing the specifics of each systematic review included in our analysis, encompassing information such as the intervention studied, the target population, outcomes assessed, comparator, the number of primary studies and participants involved, the search databases used along with their respective dates, and the effect estimates reported, such as risk ratios (RR), odds ratios (OR), hazard ratios (HR), mean difference (MD), Standardised mean difference (SMD) or similar metrics when available and their CIs, heterogeneity, publication bias, final findings, quality assessments, and a summary of the risk of bias identified in the primary studies included. A narrative approach will be used to summarize the evidence for each outcome, and we will also employ tabular formats where applicable to enhance clarity. Weightage will be given to the results of highest rated systematic review by AMSTAR-2 where the reviews show a higher level of overlap. In instances where there are discrepancies between the results of recent and high-quality systematic reviews, we will conduct a re-analysis of the primary data to arrive at a conclusion.

If newer primary studies necessitate data synthesis, a meta-analysis will be performed if more than three studies are available for each outcome. Meta-analysis will be conducted by pooling the effect size from each study using a random-effects model. The R software will be employed for the meta-analysis. Subgroup analyses will be undertaken based on variables such as participants, outcomes, or any other relevant factors that might contribute to heterogeneity. The I^2 statistic will be used to assess heterogeneity. A p-value of less than 0.05 will be deemed

For peer review only - http://bmjopen.bmj.com/site/about/guidelines.xhtml

statistically significant. Publication bias will be assessed through visual inspection of funnel plot symmetry and the Egger's test. Sensitivity analysis will be performed to determine the effect of each study on the overall result.

The quality of evidence will be determined using GRADE criteria (Grading of Recommendations, Assessment, Development, and Evaluations). Garding will be performed by considering 5 domains including risk of bias in the individual studies, inconsistency, indirectness, imprecision, and publication bias for each outcome (25). We will grade the strength of evidence (very low, low, moderate and high).

Ethics and dissemination: Ethics approval is not required for this study as it does not involve the collection of original data. The results will be disseminated through peer-reviewed publication. The findings will offer crucial insights for stakeholders, policymakers, and the general public, underlining the health implications and the role of ENDS in tobacco cessation.

Ethics approval and consent to participate: No ethical approval required

Patient consent for publication: Not required

Funding: The review is funded by World Health Organisation with registration no: 2023/1386892-0, Purchase order: 203214074.

Conflict of interest: The authors declare that they have no competing interests

Author Contributions.

Guarantor: SG. SG, JK, and AVR conceptualized the topic. AC and MS analyzed and finalized the methods. MS, AC drafted the manuscript. SG, JK, AVR, and AC reviewed, edited and proofread the final draft. SG, MS, JK, AVR, AC and finalized and approved the manuscript.

REFERENCES

1. DHHS U. E-cigarette use among youth and young adults A report of the Surgeon General. Atlanta, GA: US DHHS, CDC. National Center for Chronic Disease Prevention and Health Promotion, Office on Smoking and Health. 2016.
2. de Jong WH, Panagiotakos DB, Proykova A, Samaras T, Clemens MW, De Jong D, et al. Scientific Committee on Health, Environmental and Emerging Risks (SCHEER): final opinion on the safety of breast implants in relation to anaplastic large cell lymphoma. 2021.
3. National Academies of Sciences E, Medicine. Public health consequences of e-cigarettes. 2018.
4. Boseley S. Hon Lik invented the e-cigarette to quit smoking–but now he's a dual user. The Guardian. 2015;19.
5. Hajek P, Przulj D, Phillips A, Anderson R, McRobbie H. Nicotine delivery to users from cigarettes and from different types of e-cigarettes. Psychopharmacology. 2017;234:773-9.
6. MARTINS BNFL. FREQUÊNCIA GLOBAL E PERFIL EPIDEMIOLÓGICO DOS USUÁRIOS DE CIGARRO ELETRÔNICO: UMA REVISÃO SISTEMÁTICA.
7. Yoong SL, Hall A, Leonard A, McCrabb S, Wiggers J, d'Espaignet ET, et al. Prevalence of electronic nicotine delivery systems and electronic non-nicotine delivery systems in children and adolescents: a systematic review and meta-analysis. The Lancet Public Health. 2021;6(9):e661-e73.
8. Eaton DL, Alberg AJ, Goniewicz M, Leventhal A, Manautou JE, McGrath-Morrow S, et al. Committee on the Review of the Health Effects of Electronic Nicotine Delivery Systems. Public

For peer review only - http://bmjopen.bmj.com/site/about/guidelines.xhtml

health consequences of e-cigarettes Consensus study report for the US Food and Drug Administration Washington (DC): US National Academies of Science, Medicine and Engineering. 2018.
9. Goniewicz ML, Smith DM, Edwards KC, Blount BC, Caldwell KL, Feng J, et al. Comparison of nicotine and toxicant exposure in users of electronic cigarettes and combustible cigarettes. JAMA network open. 2018;1(8):e185937-e.
10. McRobbie H, Bullen C, Hartmann-Boyce J, Hajek P. Electronic cigarettes for smoking cessation and reduction. Cochrane database of systematic reviews. 2014(12).
11. Bravo-Gutiérrez OA, Falfán-Valencia R, Ramírez-Venegas A, Sansores RH, Ponciano-Rodríguez G, Pérez-Rubio G. Lung damage caused by heated tobacco products and electronic nicotine delivery systems: a systematic review. International journal of environmental research and public health. 2021;18(8):4079.
12. Tzortzi A, Kapetanstrataki M, Evangelopoulou V, Behrakis P. A systematic literature review of e-cigarette-related illness and injury: not just for the respirologist. International journal of environmental research and public health. 2020;17(7):2248.
13. Yang I, Sandeep S, Rodriguez J. The oral health impact of electronic cigarette use: a systematic review. Critical reviews in toxicology. 2020;50(2):97-127.
14. Li X, Zhang Y, Zhang R, Chen F, Shao L, Zhang L. Association between e-cigarettes and asthma in adolescents: a systematic review and meta-analysis. American Journal of Preventive Medicine. 2022;62(6):953-60.
15. Jonas AM, Raj R. Vaping-related acute parenchymal lung injury: a systematic review. Chest. 2020;158(4):1555-65.
16. Kalkhoran S, Glantz SA. E-cigarettes and smoking cessation in real-world and clinical settings: a systematic review and meta-analysis. The Lancet Respiratory Medicine. 2016;4(2):116-28.
17. Skotsimara G, Antonopoulos AS, Oikonomou E, Siasos G, Ioakeimidis N, Tsalamandris S, et al. Cardiovascular effects of electronic cigarettes: a systematic review and meta-analysis. European journal of preventive cardiology. 2019;26(11):1219-28.
18. Kennedy CD, van Schalkwyk MC, McKee M, Pisinger C. The cardiovascular effects of electronic cigarettes: a systematic review of experimental studies. Preventive medicine. 2019;127:105770.
19. O'Brien D, Long J, Quigley J, Lee C, McCarthy A, Kavanagh P. Association between electronic cigarette use and tobacco cigarette smoking initiation in adolescents: a systematic review and meta-analysis. BMC Public Health. 2021;21(1):1-10.
20. Aromataris E, Fernandez R, Godfrey CM, Holly C, Khalil H, Tungpunkom P. Summarizing systematic reviews: methodological development, conduct and reporting of an umbrella review approach. JBI Evidence Implementation. 2015;13(3):132-40.
21. Shamseer L, Moher D, Clarke M, Ghersi D, Liberati A, Petticrew M, et al. Preferred reporting items for systematic review and meta-analysis protocols (PRISMA-P) 2015: elaboration and explanation. Bmj. 2015;349.
22. Shea BJ, Reeves BC, Wells G, Thuku M, Hamel C, Moran J, et al. AMSTAR 2: a critical appraisal tool for systematic reviews that include randomised or non-randomised studies of healthcare interventions, or both. bmj. 2017;358.
23. Pieper D, Antoine S-L, Mathes T, Neugebauer EA, Eikermann M. Systematic review finds overlapping reviews were not mentioned in every other overview. Journal of clinical epidemiology. 2014;67(4):368-75.
24. Hennessy EA, Johnson BT. Examining overlap of included studies in meta-reviews: Guidance for using the corrected covered area index. Research synthesis methods. 2020;11(1):134-45.
25. Langendam MW, Akl EA, Dahm P, Glasziou P, Guyatt G, Schünemann HJ. Assessing and presenting summaries of evidence in Cochrane Reviews. Systematic reviews. 2013;2(1):1-9.

Tables

For peer review only - http://bmjopen.bmj.com/site/about/guidelines.xhtml

Table 1: PICO

Table 2: Search strategy for PubMed

Supplementary table 1: PRISMA-P

Supplementary table 2: Pre-piloted data extraction form

For peer review only

For peer review only - http://bmjopen.bmj.com/site/about/guidelines.xhtml

Supplementary materials

Supplementary table 1: PRISMA-P

Section and topic	Item No	Checklist item	Location
**ADMINISTRATIVE INFORMATION**			
Title:			
Identification	1a	Identify the report as a protocol of a systematic review	Page 1 (Umbrella review)
Update	1b	If the protocol is for an update of a previous systematic review, identify as such	N/A
Registration	2	If registered, provide the name of the registry (such as PROSPERO) and registration number	Page 4*
Authors:			
Contact	3a	Provide name, institutional affiliation, e-mail address of all protocol authors; provide physical mailing address of corresponding author	Page 1
Contributions	3b	Describe contributions of protocol authors and identify the guarantor of the review	Page 7
Amendments	4	If the protocol represents an amendment of a previously completed or published protocol, identify as such and list changes; otherwise, state plan for documenting important protocol amendments	Page 4
Support:			
Sources	5a	Indicate sources of financial or other support for the review	Page 7
Sponsor	5b	Provide name for the review funder and/or sponsor	Page 7
Role of sponsor or funder	5c	Describe roles of funder(s), sponsor(s), and/or institution(s), if any, in developing the protocol	Page 7
**INTRODUCTION**			
Rationale	6	Describe the rationale for the review in the context of what is already known	Page 3
Objectives	7	Provide an explicit statement of the question(s) the review will address with reference to participants, interventions, comparators, and outcomes (PICO)	Page 3,4
**METHODS**			
Eligibility criteria	8	Specify the study characteristics (such as PICO, study design, setting, time frame) and report characteristics (such as years considered, language, publication status) to be used as criteria for eligibility for the review	Page 4,5, table 1
Information sources	9	Describe all intended information sources (such as electronic databases, contact with study authors, trial registers or other grey literature sources) with planned dates of coverage	Page 5
Search strategy	10	Present draft of search strategy to be used for at least one electronic database, including planned limits, such that it could be repeated	Table 2
Study records:			
Data management	11a	Describe the mechanism(s) that will be used to manage records and data throughout the review	Page 7

For peer review only - http://bmjopen.bmj.com/site/about/guidelines.xhtml

Selection process	11b	State the process that will be used for selecting studies (such as two independent reviewers) through each phase of the review (that is, screening, eligibility and inclusion in meta-analysis)	Page 7
Data collection process	11c	Describe planned method of extracting data from reports (such as piloting forms, done independently, in duplicate), any processes for obtaining and confirming data from investigators	Page 7 Supplementary table 2
Data items	12	List and define all variables for which data will be sought (such as PICO items, funding sources), any pre-planned data assumptions and simplifications	Page 7 Supplementary table 2
Outcomes and prioritization	13	List and define all outcomes for which data will be sought, including prioritization of main and additional outcomes, with rationale	Page 7
Risk of bias in individual studies	14	Describe anticipated methods for assessing risk of bias of individual studies, including whether this will be done at the outcome or study level, or both; state how this information will be used in data synthesis	Page 8
Data synthesis	15a	Describe criteria under which study data will be quantitatively synthesized	Page 8
	15b	If data are appropriate for quantitative synthesis, describe planned summary measures, methods of handling data and methods of combining data from studies, including any planned exploration of consistency (such as I^2, Kendall's τ)	Page 78
	15c	Describe any proposed additional analyses (such as sensitivity or subgroup analyses, meta-regression)	Page 78
	15d	If quantitative synthesis is not appropriate, describe the type of summary planned	Page 8
Meta-bias(es)	16	Specify any planned assessment of meta-bias(es) (such as publication bias across studies, selective reporting within studies)	Page 8
Confidence in cumulative evidence	17	Describe how the strength of the body of evidence will be assessed (such as GRADE)	Page 9

*Pending

For peer review only

Supplementary table 2: pre-piloted data extraction form

Doi	
Author and Year	
Objectives	
Details of sources searched	
Type of included studies	

For peer review only - http://bmjopen.bmj.com/site/about/guidelines.xhtml

No of included studies	
Country of included studies	
Participants (characteristics/total number)	
Description of Intervention	
Range (years) of included studies	
Quality assessment tool used	
Quality assessment rating	
Outcomes assessed	
Results /findings	
Meta-analysis performed	
Effect size, CI, P-value of meta-analysis of main outcomes	
Heterogeneity test and results	
GRADE	
Funding	
Comments	

For peer review only

PRISMA-P checklist

Section and topic	Item No	Checklist item	Location
**ADMINISTRATIVE INFORMATION**			
Title:			
Identification	1a	Identify the report as a protocol of a systematic review	Page 1 (Umbrella review)
Update	1b	If the protocol is for an update of a previous systematic review, identify as such	N/A
Registration	2	If registered, provide the name of the registry (such as PROSPERO) and registration number	Page 4*
Authors:			
Contact	3a	Provide name, institutional affiliation, e-mail address of all protocol authors; provide physical mailing address of corresponding author	Page 1
Contributions	3b	Describe contributions of protocol authors and identify the guarantor of the review	Page 7
Amendments	4	If the protocol represents an amendment of a previously completed or published protocol, identify as such and list changes; otherwise, state plan for documenting important protocol amendments	Page 4
Support:			
Sources	5a	Indicate sources of financial or other support for the review	Page 7
Sponsor	5b	Provide name for the review funder and/or sponsor	Page 7
Role of sponsor or funder	5c	Describe roles of funder(s), sponsor(s), and/or institution(s), if any, in developing the protocol	Page 7
**INTRODUCTION**			
Rationale	6	Describe the rationale for the review in the context of what is already known	Page 3
Objectives	7	Provide an explicit statement of the question(s) the review will address with reference to participants, interventions, comparators, and outcomes (PICO)	Page 3,4
**METHODS**			
Eligibility criteria	8	Specify the study characteristics (such as PICO, study design, setting, time frame) and report characteristics (such as years considered, language, publication status) to be used as criteria for eligibility for the review	Page 4,5, table 1
Information sources	9	Describe all intended information sources (such as electronic databases, contact with study authors, trial registers or other grey literature sources) with planned dates of coverage	Page 5
Search strategy	10	Present draft of search strategy to be used for at least one electronic database, including planned limits, such that it could be repeated	Table 2
Study records:			
Data management	11a	Describe the mechanism(s) that will be used to manage records and data throughout the review	Page 7

For peer review only

Selection process	11b	State the process that will be used for selecting studies (such as two independent reviewers) through each phase of the review (that is, screening, eligibility and inclusion in meta-analysis)	Page 7
Data collection process	11c	Describe planned method of extracting data from reports (such as piloting forms, done independently, in duplicate), any processes for obtaining and confirming data from investigators	Page 7 Supplementary table 2
Data items	12	List and define all variables for which data will be sought (such as PICO items, funding sources), any pre-planned data assumptions and simplifications	Page 7 Supplementary table 2
Outcomes and prioritization	13	List and define all outcomes for which data will be sought, including prioritization of main and additional outcomes, with rationale	Page 7
Risk of bias in individual studies	14	Describe anticipated methods for assessing risk of bias of individual studies, including whether this will be done at the outcome or study level, or both; state how this information will be used in data synthesis	Page 8
Data synthesis	15a	Describe criteria under which study data will be quantitatively synthesized	Page 8
	15b	If data are appropriate for quantitative synthesis, describe planned summary measures, methods of handling data and methods of combining data from studies, including any planned exploration of consistency (such as I^2, Kendall's τ)	Page 78
	15c	Describe any proposed additional analyses (such as sensitivity or subgroup analyses, meta-regression)	Page 78
	15d	If quantitative synthesis is not appropriate, describe the type of summary planned	Page 8
Meta-bias(es)	16	Specify any planned assessment of meta-bias(es) (such as publication bias across studies, selective reporting within studies)	Page 8
Confidence in cumulative evidence	17	Describe how the strength of the body of evidence will be assessed (such as GRADE)	Page 9

For peer review only
